# Adherence among Italian paediatricians to the Italian guidelines for the management of fever in children: a cross sectional survey

**DOI:** 10.1186/1471-2431-13-210

**Published:** 2013-12-18

**Authors:** Elena Chiappini, Sofia D’Elios, Rachele Mazzantini, Paolo Becherucci, Monica Pierattelli, Luisa Galli, Maurizio de Martino

**Affiliations:** 1 Department of Health Sciences, Paediatric Section, Anna Meyer Children’s University Hospital, Florence, Italy; 2 Primary Care Paediatrician, Florence, Italy

## Abstract

**Background:**

Italian guidelines for the management of fever in children (IFG) have been published in 2009 and thereafter disseminated in all country. A survey was conducted before their publication and three years later to investigate their impact on knowledge and behaviors of paediatricians.

**Methods:**

A questionnaire was administered to convenient samples of paediatricians in 2009 and in 2012, eliciting information about fever definition, methods of temperature measurement, and antipyretic use. Differences in responses between 2009 and 2012 and between paediatricians who were or were not aware of the IFG were evaluated.

**Results:**

The responses rates were 74% (480/648) in 2009 and 69% (300/434) in 2012. In 2012 168/300 (56%) of participants were aware of the IFG. The proportion of paediatricians who correctly would never suggest the use of physical methods increased from 18.7% to 36.4% (P < 0.001). In 2009 11% of paediatricians declared that the use of antipyretic drugs depends on patient discomfort and did not use a temperature cut off. In 2012 this percentage reached 45.3% (P < 0.001). Alternate use of antipyretics decreased from 27.0% to 11.3% (P < 0.001). Use of rectal administration of antipyretics in absence of vomiting decreased from 43.8% in 2009 to 25.3% in 2012 (P < 0.001). In general, improvements were more striking in paediatricians who were aware of the IFG than in those who were not aware of them.

**Conclusions:**

Behaviours of Italian paediatricians improved over time. However, some wrong attitudes need to be further discouraged, including use of physical methods and misuse of rectal administration. Further strategy to disseminate the IFG could be needed.

## Background

Fever is a chief complaint in children undergoing pediatric evaluation, but the attitude in the management of this sign largely varies among paediatricians [1]. Guidelines for the management of fever in children have been published in many Western countries but the gap between available evidence and clinical practice seems still to be substantial and poor adherence to the guideline recommendations has been reported [2]. The Italian Fever Guidelines (IFG) have been issued by the Italian Society of Pediatrics in 2009 [1]. The IFG aim was to give recommendations for the correct body temperature measurement, management of fever/elevated temperature in children and did not cover diagnostic and therapeutic issues in febrile children. The guideline was developed according to methods accepted by the National Guideline Program (NGLP), a joint effort of the Italian Health Ministry and the National Health Institute that is aimed at promoting a high quality of care in the National Health Service [3]. In particular, the IFG are evidence based guidelines, and the methodology is very similar to that one adopted in other similar European and UK Guidelines (i.e. the NICE guidelines) [4-6]. These guidelines have been widely disseminated through a variety of strategies, including numerous national and local conferences, websites, courses for primary care and hospital paediatricians (Additional file 1: Appendix 1). Therefore it was important to investigate the actual impact of IFG on the clinical practice after their large dissemination, aimed to improve the management of fever in children and to standardize the behavior of physicians. In this survey the same questionnaire was administered to convenient samples of Italian pediatricians before and three years after the publication of the IFG, in order to investigate the impact of IFG on the clinical practice.

## Methods

### Survey given to paediatricians

All subjects were interviewed by the use of a standardized self-administered questionnaire, designed on the bases of both previous similar surveys [7,8] and on the recent United States, UK and Italian Guidelines for the management of fever in children [1,4-6]. The questionnaire elicited information about definition and measurement of fever and antipyretic management [7-9]. All the sixteen close ended questions are reported in Additional file 1: Appendix 2. All responses remained anonymous, and no identifier could be used to trace the participants on the survey.

The questionnaire was firstly administered to all the paediatricians attending the 14^th^ Italian National Congress of Practice Paediatrics, held in Florence in November 2009, before the publication of the IFG [1]. The results of this first survey have been reported in a previous study [10]. The IFG have been published in 2009 and have been also spread through 6 publications in Italian Journals, 2 publications in International Journals, 25 oral communications in National Conferences, 28 web sites, 7 courses for physicians and medicine students (Additional file 1: Appendix 1). Our hypothesis was that the IFG dissemination would have improved the behaviours of paediatricians who were aware of them, but not of those who were not aware.

The same questionnaire was administered to the paediatricians attending the 12^th^ National Congress of Italian Society of Pediatric Infectious Diseases, held in Florence in 2012, three years after the first survey. Participants were also asked whether they were aware of the IFG and whether they already participated in the 2009 survey. These surveys were not commercially sponsored. This study was approved by the ethics committee of the Anna Meyer Children’s University Hospital of Florence, Italy.

### Statistical analysis

The results were given as absolute numbers and percentages. The percentages of responses to the questions have been calculated on the total of the participants. Differences in responses between the 2009 and the 2012 surveys were evaluated by contingency table analysis with the ×^2^ or the Fisher’s exact test (2 grades of freedom), as appropriate. Relatively to the 2012 survey, the same analysis was applied to evaluate the differences in responses between paediatricians who were or were not aware of the IFG [1]. SPSS software package (SPSS 11.5; Chicago, IL) was used, and p < 0.05 was considered as statistically significant.

## Results

Among paediatricians attending the two paediatric conferences, the responses rates were 74% (480/648) in 2009 and 69% (300/434) in 2012 (P = 0.531); 38/300 (12.6%) of the paediatricians enrolled in 2012 survey declared to have previously participated in the 2009 survey, 95% of them (36/38) were aware of the IFG. In 2012 168/300 (56%) participants were aware of the IFG. The most of participants in 2009 survey were family paediatricians (93%, 446/480), while in 2012 survey all classes were represented, as described in Table 1. Participants’ demographic data were not collected.

**Table 1:** **Participants category in the 2009** (**n** = **480**) **and**/**or in the 2012 surveys** (**n** = **300**)

	**Participants in 2009 survey n (%)**	**Participants in 2012 survey n (%)**	**Participants in 2012 survey who were aware of the IFG n (%)**	**Participants in 2012 survey who were not aware of the IFG n (%)**
Hospital paediatricians	14 (3.0)	85 (28.3)	55 (32.7)	30 (22.7)
Family paediatricians	446 (93.0)	134 (44.7)	78 (46.4)	56 (42.4)
Residents in paediatrics	0 (0.0)	57 (19.0)	24 (14.3)	33 (25.0)
Other	20 (4.0)	24 (8.0)	11 (6.6)	13 (9.9)
Total	480 (100.0)	300 (100.0)	168 (100.0)	132 (100.0)

### Definition of fever and methods for body temperature measurement

Although the body temperature varies even within the same individual and is influenced by circadian rhythm, physical activity, and other factors, from a practical point of view both the World Health Organization and the IFG recommend to consider fever as body temperature above 37.5°C. [1,6]. Correctly, the percentage of paediatricians who regarded as fever a body temperature above 37.5°C was 32.7% (157/480) in 2009, and this increased in 2012 (44.7%; 134/300; P = 0.001). On the other hand the paediatricians who, wrongly, considered fever a body temperature above 38°C decreased from 41.2% to 29.7% (198/480 *vs*. 89/300; P = 0.001).

Axillary temperature measurement using a digital thermometer is recommended in children younger than 4 weeks. In the hospital or ambulatory care setting, axillary temperature measurement using a digital or infrared thermometer (i.e. tympanic thermometer) is recommended in children older than 4 weeks while Axillary temperature measurement using a digital thermometer is recommended when the measurement is executed by parents/caregivers [1,6]. Uncorrectly, in 2009 survey, most of the physicians (64.4%) recommended to measure the body temperature rectally in children aged <1 year. In 2012 these percentage decreased (P < 0.0001) and this decrease was more striking among paediatricians who were aware than in those who were not aware of the IFG (P = 0.035) (Table 2).

**Table 2:** **Temperature monitoring methods used by paediatricians who participated in the 2009** (**n** = **480**) **and**/**or in the 2012 surveys** (**n** = **300**) **and**/**or were aware** (**n** = **168**) **or not aware** (**n** = **132**) **of the IFG**

	**Participants in 2009 survey n (%)**	**Participants in 2012 survey n (%)**	**P**	**Participants in 2012 survey who were aware of the IFG n (%)**	**Participants in 2012 survey who were not aware of the IFG n (%)**	**P**
Site/mode of measurement in children under one year						
Axillary	111 (23.1)	118 (39.3)	≤0.0001	83 (49.4)	35 (26.5)	0.000
Rectal	309 (64.4)	124 (41.3)	≤0.0001	60 (35.7)	64 (48.4)	0.035
Groin crease	51 (10.7)	38 (12.7)	0.299	17 (10.1)	21 (15.9)	0.186
Oral	1 (0.2)	0 (0)	0.812	0 (0)	0 (0)	
Auricular	4 (0.8)	18 (6.0)	0.000	6 (3.6)	12 (9.1)	0.080
On the forehead	4 (0.8)	2 (0.6)	0.871	2 (1.2)	0 (0)	0.587
Site/mode of measurement in children over one year						
Axillary	388 (80.8)	242 (80.7)	0.994	141 (83.9)	101 (76.5)	0.142
Rectal	39 (8.1)	9 (3.0)	0.006	3 (1.8)	6 (4.5)	0.294
Groin crease	32 (6.7)	15 (5.0)	0.425	8 (4.8)	7 (5.3)	0.957
Oral	0 (0)	2 (0.7)	0.288	0 (0)	2 (1.5)	0.376
Auricular	9 (1.9)	29 (9.6)	0.000	13 (7.7)	16 (12.1)	0.281
On the forehead	11 (2.3)	3 (1.0)	0.296	3 (1.8)	0 (0)	0.338

Paediatricians who correctly recommended an in the axilla measurement of fever, in children aged <1 year, increased from 23,1% in 2009 to 39.3% in 2012 (P < 0.001) (Table 2).

The proportion of paediatricians who recommended an in the axilla measurement of fever in older children was similar in 2009 and 2012 survey (P = 0.994). In 2012 an increase in the proportion of paediatricians who used the auricular measurement in children over 1 years of age was observed (P < 0.001). The most commonly type of thermometer used was the digital thermometer in both groups (P = 0.203). Curiously, in 2012 16.7% (50/300) of paediatricians still recommended the use of the mercury in glass thermometer, even if this has been withdrawn from the market (Table 3). Likely they would suggest the use of the mercury in glass thermometer whether the family still owns an old device.

**Table 3:** **Type of thermometer recommended and use of tympanic thermometer by paediatricians who participated in the 2009** (**n** = **480**) **and**/**or in the 2012 surveys** (**n** = **300**) **and who were aware** (**n** = **168**) **or not aware** (**n** = **132**) **of the IFG**

	**Participants in 2009 survey n (%)**	**Participants in 2012 survey n (%)**	**P**	**Participants in 2012 survey who were aware of the IFG n (%)**	**Participants in 2012 survey who were not aware of the IFG n (%)**	**P**
Type of thermometer recommended						
Digital*	305 (63.5)	203 (67.7)	0.203	131 (78.0)	72 (54.5)	0.000
Auricular	10 (2.0)	15 (5.0)	0.041	6 (3.6)	9 (6.8)	0.311
Other	55 (11.8)	32 (10.6)		13 (7.7)	19 (14.4)	
Tympanic infrared thermometer used						
Hospital care setting*	232 (48.3)	203 (67.7)	≤0.0001	127 (75.6)	76 (57.6)	0.001
Home setting	34 (7.1)	9 (3)	0.023	1 (0.6)	8 (6.1)	0.016
Both	174 (36.2)	300 (29.3)	0.056	40 (23.8)	48 (36.4)	0.000

Note: (*) right answer.

Other: skin infrared, plastic strip placed on forehead, dummy-pacifier style, no thermometer recommended.

**Table 4:** **Differences in antipyretics use in participants in the 2009** (**n** = **480**) **and**/**or in the 2012** (**n** = **300**) **survey**, **and who were aware** (**n** = **168**) **or not aware** (**n** = **132**) **of the IFG**

	**Participants in 2009 survey n (%)**	**Participants in 2012 survey n (%)**	**P**	**Participants in 2012 survey who were aware of the IFG n (%)**	**Participants in 2012 survey who were not aware of the IFG n (%)**	**P**
First choice antipyretic drug						
Acetaminophene	463 (96.4)	295 (98.3)	0.188	167 (99.4)	128 (97.0)	0.23
Ibuprofene	17 (3.6)	4 (1.3)	0.104	0	4 (3.0)	0.083
Aspirin	0 (0.0)	0 (0.0)		0	0	
Other (metamizole. betamethasone)	0 (0.0)	1 (0.4)	0.812	1 (0.6)	0	0.904
Second choice antipyretic drug						
Acetaminophene	32 (6.7)	19 (6.3)	0.973	7 (4.2)	12 (9.1)	0.134
Ibuprofene	440 (91.6)	276 (91.0)	0.975	159 (94.6)	117 (88.6)	0.091
Aspirin	1 (0.3)	2 (0.7)	0.681	0 (0.0)	2 (1.5)	0.376
Other (metamizole. betamethasone)	7 (1.4)	3 (1.0)	0.821	2 (1.2)	1 (0.8)	0.833
Alternating use of antipyretic drugs and use of atipyretic according to child’s discomfort						
Yes	130 (27.0)	34 (11.3)	≤0.0001	12 (7.0)	22 (17.0)	0.016
No	350 (72.9)	266 (88.7)	≤0.0001	156 (93.0)	110 (83.0)	0.000
Use of physical methods						
With antipyretic drug	62 (13.0)	29 (9.6)	0.207	11 (6.5)	18 (14)	0.062
Before the antipyretic drug	15 (3.3)	9 (3.0)	0.909	2 (1.2)	7 (5.0)	0.083
If the fever persists	315 (65.0)	153 (51.0)	≤0.0001	71 (42.3)	82 (62.0)	0.000
No never	88 (18.7)	109 (36.4)	≤0.0001	84 (50.0)	25 (19.0)	0.000

Correctly, in children > 1 year of age a tympanic measurement based on the infrared thermometer was recommended in the hospital care setting by 48.3% of participants in 2009, while this proportion increased to 67.7% in 2012 (P < 0.001). This increase was more evident in paediatricians who were aware of the IFG than in those were not aware of the IFG (P = 0.001). In 2012 the percentage of pediatricians who, incorrectly, recommended the use of infrared tympanic thermometer by parents decreased (P = 0.023), and of these most were not aware of the IFG (P = 0.016) (Table 3).

### Management of febrile children

#### Information given to parents and caregivers

In 2009 and in 2012 the most of participants declared to give written prescription regarding modes and administration of antipyretic drugs to the parents/caregivers (61.2%; 294/480, in 2009; *vs*. 55.3%; 166/300, in 2012; P = 0.119). The information was commonly provided to the parents/caregivers within three months of age, on the occasion of his/her first paediatric assessment or first immunization (83.6%; 401/480 in 2009 *vs*. 81.1%; 243/300; P = 0.416 ).

#### Physical methods

The IFG discourage the use of physical methods, as sponging or ice pack, to reduce the body temperature in children [1]. In both surveys most of paediatricians recommended the use of physical methods if fever persisted over time (65.0%; 315/480 *vs*. 51.0%; 153/300; P < 0.001). In 2012 most of those declaring to adopt this behaviour were not aware of the IFG (42.3%; 71/168 *vs*. 62.0%; 82/132; P < 0.001).

The proportion of paediatricians who would never suggest the use of physical methods increased from 18.7% (88/480) in 2009 to 36.4% (109/300) in 2012, (P < 0.001). This increase was more striking in paediatricians who were aware the IFG (50%; 84/168 *vs*. 19%; 25/132; P < 0.001).

#### Antipyretic drugs

In 2009 only 11% (56/480) of paediatricians, correctly, declared that there wasn’t a temperature cut off to recommend the use of antipyretics, but this depends on the patient’s discomfort; while in 2012 a greater percentage of paediatricians (45.3%; 136/300; P < 0.001) declared it and 62.5% (85/136) of them were aware of the IFG (Table 4).

In both surveys paracetamol was the first choice antipyretic (P = 0.188) and ibuprofen was the second choice drug (P = 0.975) (Table 4). No paediatrician declared to use acetylsalicylic acid or steroids as first choice drug, but, worrisomely, 1.7% (8/480 *v*s. 5/300; P = 0.744) of paediatricians declared to use them as possible second choice drugs, both in 2009 an in 2012.

Contrary to the IFG recommendations, in 2009 the 27.0% (130/480) of participants declared to recommend the alternate use of ibuprofen and paracetamol. This proportion decreased to 11.3% (34/300) in 2012 (P < 0.001) and most of them (65%; 22/34) included paediatricians who were not aware of the IFG.

In 2009 and 2012, correctly, most paediatricians preferred oral to rectal administration of paracetamol (73.1% 351/480 in 2009 *vs*. 83.0% 249/300 in 2012; P = 0.002).

Correctly in 2009, 56.2% (270/480) of paediatricians suggested the rectal administration only in the presence of vomiting and in 2012 this proportion increased to 74.7% (224/300; P < 0.001); this increase was more striking in paediatricians who were aware of the IFG than in those who not were aware of them (81.5%; 137/168 *vs*. 66%; 87/132; P < 0.003). However, in 2009 24.3% (117/480) of paediatricians declared to prefer rectal administration because it was considered to be more practical, while in 2012 only 12.0% (36/300; P < 0.001) declared it and 69.4% (25/36) of them were not aware of the IFG.

Incorrectly, both in 2009 and in 2012 about 50.0% (240/480, 150/300; P = 0.638) of the paediatricians reported to use a higher pro-Kg dosage of paracetamol when it is administered rectally.

In 2009, contrary to the guidelines recommendations, use of paracetamol or ibuprofen was recommended for the prevention of febrile convulsion in febrile children by 60.6% (291/480) of paediatricians. While, in 2012, this percentage decreased to 40.6% (122/300) and 64.8% (79/122) of them were not aware of the IFG.

## Discussion

Guidelines should be not only issued, but also well disseminated in order to facilitate their adoption and actually to obtain changes in clinical behaviours and attitudes in the real settings [1,4-6]. Indeed, it has been previously reported that several guideline recommendations have been poorly incorporated into the clinical practice after their development and distribution [2,11]. In our study 56% of participants in 2012 survey are aware of the IFG. Interestingly about 95% of those who participated in the 2009 survey were aware of the IFG. We can speculate that this finding could be due to the interest aroused after the execution of the 2009 questionnaire. A survey conducted by Grol *et al*. among a random sample of 10% (453) of all family physicians in Netherlands, approximately 2 years after publication of the first set of guidelines, showed most physicians to be well informed about practice guidelines, in general (only 7% did not know about them) [12]. On the contrary, Flores G. *et al*. conducted a cross-sectional survey to determine practice guidelines attitudes, beliefs and practices among US paediatricians. A list of 2000 randomly-selected members of the American Academy of Pediatric who resided in United State was obtained and a survey and self-addressed stamped envelope were mailed to each subject. Practice guidelines were used by 35% of participants, in particular, only 9% of them declare to adopt fever guidelines [13].

Many studies investigated the barriers to the implementation of CPG in healthcare and the effective strategies for translating research into practice, however it is recognized that identification of local barriers to change is pivotal to changing practioners’ behaviour toward adoption of guidelines [14]. In a systematic review by Cabana DM *et al*. illustrated variety of barriers to guideline adherence, which include lack of awareness, lack of familiarity, lack of agreement, lack of self-efficacy, lack of outcome expectancy, the inertia of previous practice, and external barriers [15]. A single implementation strategy may not be as effective as a multifaceted approach to ensure the awareness of the healthcare professionals of the existence of the guidelines, to increase their familiarity with its recommendations and to detect and address barriers to the implementation of these recommendations [14].

Our survey was aimed to evaluate the impact of the IFG on knowledge and behaviours of a sample of Italian paediatricians, through the administration of the same questionnaire before and three years after the publication of the IFG [1,10]. In 2009, we recorded a wide spread of uncorrected or dangerous practices by a large share of paediatricians, including the alternate use of antipyretics, rectal administration of drugs even in the absence of vomiting, and the use of antipyretics for the prevention of febrile convulsions. In 2012, the key messages of the IFG have been adopted by larger proportions of paediatricians [1]. In particular, the use of physical methods decreased, especially among those who claimed to be aware of the IFG, but, worrisomely, still about half of paediatricians recommend their use in some circumstances. The use of antipyretics according to the child’s discomfort and not to a particular temperature cut-off increased (11.7% in 2009 *vs*. 45.3% in 2012). The alternate use of antipyretic drugs decreased from 27.0% in 2009 to 11.3% in 2012.

Additionally, within the paediatricians participating in the 2012 survey, significant differences were found between those who were aware and those who were not aware of the IFG, suggesting that the IFG recommendations have been largely adopted. For example, the use of antipyretic rectally only in the presence of vomiting was significantly more common among paediatricians who were aware of IFG while the alternate use of antypiretic drugs and physical methods were more common among paediatricians who were not aware of IFG.

Notably, improvements in many behaviours have been observed also among paediatricians who were not aware of IFG. Our data cannot explain the reason for changes in practice in the subpopulation of paediatricians who declared to be not aware of IFG. We may speculate that discussions with colleagues, parents or others may have created a sort of “heard immunity” in this group of subjects [16].

Our findings highlight some concepts that should be further implemented. About 1% of paediatricians, worryingly, still declare to recommend steroids or acetylsalicylic acid as second choice drugs. Moreover, the message that the temperature should be measured correctly, considering the child's age and the setting should be implemented.

Other authors previously demonstrated large discrepancies between guideline recommendations and clinical practice regarding the management of the febrile child. In a Swiss study by Lava SA *et al*., the paediatricians were interviewed by a close-ended questionnaire, sent via electronic mail [17]. Consistently to our results, among Swiss paediatricians paracetamol was the first choice antipyretic drug and ibuprofen was the second choice. Most of participants prescribed alternate use of two antipyretics or physical methods to reduce the body temperature (respectively 77%, 65% respectively).

Demir *et al*. conduced in Turky a cross sectional study about knowledge, attitudes and misconceptions of primary care physicians regarding fever in children. A self-administered questionnaire was sent to 80 paediatricians working in a province with a population of 600 000 people. They demonstrated that most of the physicians (83.8%) correctly recommended an axillary measurement of fever to the parents of the febrile child. Only 26.2% of physicians took into consideration signs and symptoms other than fever (malaise, irritability, prolonged crying, signs of infection) when prescribing the antipyretic. Only 15% of physicians indicated that they prescribed antipyretics considering the child’s comfort; 78.7% of paediatricians agreed that paracetamol and ibuprofen can be used alternatively. Most of paediatricians (87.5%) indicated that physical methods should be recommended to reduce fever [18].

Our investigation may have potential limitations. Our results may not generalize to all paediatricians. Paediatricians included in the study constituted approximately 6.0% of all the paediatricians currently working in Italy [10]. Personal data (i.e, data regarding age and residence of paediatricians) were not collected. Thus, our study does not provide information regarding possible differences in responses according to the geographical provenience and age. This issue, together with the fact that distribution of physicians in categories was different in 2009 vs. 20012, could have, at least in part, influenced our results. It is well known that self-reported behaviours can be misleading since some participants might not complete the survey as carefully as they would act in real settings [19]. Participants, could be more interested in fever management than those who did not agree to participate into the study. Finally, we did not calculate our study power since we do not have data to assume a priori the expected proportions of changes in answer.

## Conclusion

In conclusion, our findings underline the importance of the IFG dissemination in order to improve the paediatricians’ knowledge about fever. Our survey suggests that some wrong behaviours need to be further discouraged, as the alternate use of antipyretics and their rectal administration in the absence of vomiting. Further strategy to disseminate the IFG via other channels and to remove possible obstacle to IFG adherence could be needed.

## Abbreviations

IFG: Italian Fever Guidelines.

## Competing interests

In the past five years, we have not received any reimbursements, fees, funding, or salary from any organization that may in any way gain or lose financially from the publication of this manuscript. There is no organization financing this manuscript (including the article processing charge). We do not hold any stocks or shares in any organization that may in any way gain or lose financially from the publication of this manuscript. There are no non-financial competing interests (political, personal, religious, ideological, academic, intellectual, commercial or any other) to declare in relation to this manuscript.

## Authors’ contributions

EC conceived the study, participated in the design and coordination, and drafted the manuscript. SD and RM performed the statistical analyses and helped draft the manuscript. PB and MP participated in the study design and helped draft the manuscript. LG and MdM helped in the conception and design of the study and helped draft the manuscript. All authors read and approved the final manuscript.

## Pre-publication history

The pre-publication history for this paper can be accessed here:

http://www.biomedcentral.com/1471-2431/13/210/prepub

## Supplementary Material

Additional file 1 **Appendix 1.** Questionnaire 2009 and 2012. **Appendix 2**: Different strategies adopted to disseminate IFG. Click here for file
