# Supplementary material for: Adherence among Italian paediatricians to the Italian guidelines for the management of fever in children: a cross sectional survey
Source: BMC Pediatr. 2013 Dec 18;13:210. doi: 10.1186/1471-2431-13-210 (PMC3878332; doi:10.1186/1471-2431-13-210)
Supplement: Additional file 1 — Appendix 1. Questionnaire 2009 and 2012. Appendix 2: Different strategies adopted to disseminate IFG. [file 1471-2431-13-210-S1.doc]

Appendix 1

Questionnaire 2009 and 2012

1. Where should body temperature be measured in children under one year?

a) the armpit

b) the rectum

c) groin crease

d) the mouth

e) the ear

f) on the forehead

2. Where should the body temperature be measured in children over one year?

a) the armpit

b) the rectum

c) groin crease

d) the mouth

e) the ear

f) on the forehead

3. What kind of thermometer do you suggest to measure temperature?

a) mercury-in-glass

b) electronic

c) auricular

d) skin infrared

e) plastic strip placed on forehead

f) “dummy”

g) I don’t suggest any particular thermometer

4. Tympanic infrared thermometer must be used:

a) by skilled labours in the hospital/ambulatory setting

b) by parents at home

c) in both situations

5. Over what temperature do you consider that a child has fever?

a) 36.5 °C

b) 37 °C

c) 37.5 °C

d) 38 °C

e) 38.5 °C

f ) 39 °C

6. Above what temperature do you administer antipyretics?

a) <37 °C

b) 37.5 °C

c) 38 °C

d) 38.5 °C

e) 39 °C

f) not exist a temperature cut off, depend on patient malaise

7. Which antipyretic drugs do you usually suggest to use?

a) acetaminophen

b) ibuprofen

c) aspirin

d) other (metamizole,betamethasone)

8. Which other drugs do you suggest in addition to the previous?

a) acetaminophen

b) ibuprofen

c) aspirin

d) other (metamizole,betamethasone)

9. When the temperature is not going down quickly, do you believe it is useful to associate two or more antipyretic drugs?

a) yes

b) no

10. Do you suggest to use physical methods as sponging or ice pack to reduce a child’s body temperature?

a) yes, with the antipyretic drug

b) yes, before the antipyretic drug

c) only if the temperature is not going down after the antipyretic drug

d) no, never

11. How do you suggest to administer antipyretic drug?

a) orally

b) rectally

12. If so, why do you suggest to administer antipyretic drug rectally?

a) it’s more useful

b) it’s more practical

c) because parents prefer this way

d) only in the presence of vomiting

13. Do you suggest a higher dose of antipyretic drug when you administer it rectally?

a) yes

b) no

14. When do you give preventive information about fever management?

a) at the first medical examination of the newborn

b) at the first vaccinations

c) six months’ examination

d) one year’s examination

e) I don’t give any preventive information about fever management

15. Do you give a written prescription regarding modes and administration of antipyretic drugs?

a) yes

b) no

c) often

d) rarely

e) only for patients who have difficulty in comprehension

16. Do you think that antipyretics should be used to prevent febrile convulsions in children?

a) yes

b) no

Appendix 2

Different strategies adopted to disseminate IFG

*Publications in Italian Journals*

- de Martino M, Principi N. Febbre nel bambino: dalle conoscenze biologiche, le basi per la migliore gestione clinica, Linee Guida della Società Italiana di Pediatria. Prospettive in Pediatria 2008;38:284-291.
- de Martino M, Principi N. [Gestione del segno/sintomo febbre in pediatria: linee guida della Società Italiana di Pediatria](http://www.minervamedica.it/it/riviste/minerva-pediatrica/articolo.php?cod=R15Y2008N05A0489). [Minerva Pediatrica 2008;60:489-501](http://www.minervamedica.it/it/riviste/minerva-pediatrica/articolo.php?cod=R15Y2008N05A0489)
- Becherucci P, Bonsignori F, Chiappini E, de Maria A, de Martino M, Esposito S, Faldella G, Festini F, Galli L, Longhi R, Lucchesi B, Marseglia GL, Minoli L, Mugelli A, Principi N, Pecco P, Squaglia S, Tambaro P, Tovo PA, Tulimiero P, Zavarise G. Gestione del segno/sintomo febbre Area pediatrica. 2009;10:11-32.
- de Martino M, Principi N. Gestione della febbre in Pediatria. Medicinae Doctor 2011;18-20.
- Merlo AM. Clinical management of the feverish child: a summary of the Italian Society of Pediatrics Guidelines on fever in children. Italian Journal of Pediatric Nursing Science 2012; 4:19-22.
- Di Mauro G, Marinello L, Di Mauro L. Strategie “evidence based” per il controllo della febbre. Pediatria Preventiva e Sociale 2012; 1:8-13.

*Publications in International Journals*

- Chiappini E, Principi N, Longhi R, Tovo PA, Becherucci P, Bonsignori F, Esposito S, Festini F, Galli L, Lucchesi B, Mugelli A, de Martino M. Writing Committee of the Italian Pediatric Society Panel for the Management of Fever in Children. Management of fever in children: summary of the Italian Pediatric Society guidelines. Clin Ther 2009, 31:1826–1843.
- Chiappini E, Venturini E, Principi N, Longhi R, Tovo PA, Becherucci P, Bonsignori F, Esposito S, Festini F, Galli L, Lucchesi B, Mugelli A, de Martino M. Update of the 2009 italian pediatric society guidelines about management of fever in children. Clin Ther. 2012; 34:1648-1653

*Italian oral communications in Conferences*

- Fourth National Italian Conference Society of Pediatric Emergency Medicine, Catanzaro 28 ^th^ -31^st^ May, 2008.
- Twentieth Congress of the Italian Society for Preventive and Social Paediatrics, Caserta 30^th^ May-2^nd^ June, 2008.
- Second National Conference of the Italian Federation of Primary care Paediatricians (FIMP), Naples 1^st^-4^th^ October, 2008.
- 64^th^ National Congress of Italian Paediatric Society (SIP), Genoa 15^th^ -18^th^ October, 2008.
- “Opinioni a Confronto”, Pavia 9^th^ -11^th^ October, 2008.
- “La febbre e le febbri”, Catania 27^th^ March, 2009.
- FORMAT, Verona 21^st^-22^nd^ May, 2009.
- Paediatric workshop about management of fever, Lido di Camaiore (LU) 10^th^ October, 2009.
- Tuscan Workshop for primary care paediatricians, Siena 18^th^ September, 2010.
- National Congress of the Italian Federation of Primary care Paediatricians (FIMP), Florence 30 September, 1^st^ and 2^nd^ October, 2010.
- XII National Congress of the Society of Preventive and Social Pediatrics, Taormina 27^th^-29^th^ May, 2010.
- Workshop on Management of the sign / symptom of fever in children, Pistoia 25^th^ September, 2010.
- Workshop on Management of the sign / symptom of fever in children, Pisa 25^th^ September, 2010.
- Regional Paediatric Congress, Monza 22^th^ June, 2011.
- Therapeutic appropriateness fever and pain in children, Camerano (AN) 8^th^ October, 2011.
- “Opinioni a confront”, Pavia 7^th^ -8^th^ October, 2011.
- Congress of the Italian Federation of Primary care Paediatricians (FIMP), Montecatini Terme (PT) 26^th^ November, 2011.
- Congress of the Italian Federation of Primary care Paediatricians (FIMP), Catania 3^th^ -11^th^ December, 2011.
- Congress of the Italian Federation of Primary care Paediatricians (FIMP), Rome, 21^st^ April, 2012.
- FORMAT, Verona 25^th^ -26^th^ May, 2012.
- Third Congress f SIMEUP, Catanzaro 24^th^ -27^th^ May, 2012.
- National Congress of Allergology and Paediatrics, Bari 7^th^ -9^th^ June, 2012.
- Fifteenth Congress of the Italian Federation of Primary care Paediatricians (FIMP), Crotone 13^th^ -14^th^ October, 2012.
- Seventh Sicilian Congress of the Italian Federation of Primary care Paediatricians (FIMP), Palermo 26^th^ -27^th^ -28^th^ October, 2012.
- National Congress of the National Association of Medical Board, Genoa 1^st^ December, 2012.

*Websites for paediatricians or nurses*

- SICUPP www.sicupp.org/index.php/linee-guida
- SIP: www.snlg-iss.it/news_LG_SIP_febbre
- ISS: www.snlg-iss.it
- http://www.ipasvi.it/ecm/percorsi-guidati/la-febbre-nel-bambino-id11.htm
- http://ebookbrowse.com/lg-sip-febbre-aggiornamento-2011-pdf-d211822533
- storage.aicod.it/portale/ausl/Corso_guardia_medica_Drssa_Fragni.pdf
- Sito infermieristica pediatrica www.infermieristicapediatrica.it/pdf/lineeguidafebbre.pdf
- prontosoccorso.eumed.org/area.../se-il-bambino-ha-febbre/
- http://www.nonfartiinfluenzare.it/cont/influenza-login/11/farti-influenzare.asp
- http://prontosoccorso.eumed.org/area-pubblica/6001/la-gestione-della-febbre-nei-bambini/
- http://www.be-med.it/linee-guida/64-pediatria/48-linee-guida-sulla-febbre.html

*Websites for parents/caregivers*

- http://sip.it/per-i-genitori/febbe-nel-bambino-cosa-fare
- it.wikipedia.org/wiki/Febbre
- www.giovanigenitori.it › esperti › Il pediatra
- http://www.nonfartiinfluenzare.it/cont/influenza-login/11/farti-influenzare.asp
- http://donna.stage.tiscali.it/articoli/pediatra/09/04/consigli_febbre_bimbo_123.htm
- http://pediatracimino.blogspot.it/2012/12/medici-e-genitori-alle-prese-con-la.html
- http://www.intesa.unifarm.it/public/file_info/info204-la_febbre_nel_bambino.pdf
- http://www.pensiero.it/news/news.asp?IDnews=1288
- http://www.agendasalute.com/article/febbre
- http://www.infosalute.info/news.php?id=1584&rimedi-per-la-febbre-alta-nei-bambini-pimavera-e-sbalzi-di-temperatura
- http://news.paginemediche.it/it/232/comunicati-tampa/pediatria/detail_176460_primavera-e-sbalzi-di-temperatura-quando-la-febbre-e-uno-stato-di-emergenza-e-quando-non-lo-e.aspx?c1=77
- http://www.ipasvi.it/per-il-cittadino/click-salute/la-febbre-nel-bambino-id11.htm
- http://www.diggita.it/story.php?title=Gestione_del_segnosintomo_febbre_in_pediatria_Linee_Guida_della_Societ%C3%A0_Italiana_di_Pediatria
- http://oknotizie.virgilio.it/info/71f112d48e34e14f/4pediatriapa_gestione_del_segno_sintomo_febbre_in_pediatria_linee_guida_della_societa_italiana_di_pediatria.html
- http://www.repubblica.it/salute/medicina/2012/12/04/news/febbre_antipiretico_bambini-48063989/
- http://lifestyle.tiscali.it/socialnews/mamma_bimbo/Minelli/5450/articoli/Come-gestire-la-febbre.html
- www.aslto5.piemonte.it/allegato.aspx?

*Courses for physicians and medicine students*

- “Quanta febbre hai? Come misurare la temperatura corporea ai bambini senza il termometro al mercurio” . Annual Training Program of the University Hospital Anna Meyer, Florence, 15^th^ November 2011
- Annual course for medical students, University of Florence
- Course of Federation of Italian Pharmacists Orders First edition, June 2009
- Course “Fata Junior”, Novembre 2011
- http://blogpinali.wordpress.com/2011/02/05/linee-guida-sulla-febbre-in-pediatria/
- storage.aicod.it/portale/ausl/Corso_guardia_medica_Drssa_Fragni.pdf
- http://www.axadacatania.com/studenti-medicina/linee-guida/pediatria/
